# Phenotypic and genetic features of a novel clinically isolated rough morphotype *Candida auris*

**DOI:** 10.3389/fmicb.2023.1174878

**Published:** 2023-06-07

**Authors:** Sufei Tian, Jian Bing, Yunzhuo Chu, Hailong Li, Qihui Wang, Shitong Cheng, Jingjing Chen, Hong Shang

**Affiliations:** ^1^National Clinical Research Center for Laboratory Medicine, Department of Laboratory Medicine, The First Hospital of China Medical University, Shenyang, China; ^2^State Key Laboratory of Genetic Engineering, School of Life Sciences, Fudan University, Shanghai, China

**Keywords:** *Candida auris*, rough morphotype, RNA-seq, *ACE2*, *PSA31*

## Abstract

**Introduction:**

*Candida auris* is a newly emerging pathogenic fungus of global concern and has been defined by the World Health Organization (WHO) as a member of the critical group of the most health-threatening fungi.

**Methods:**

This study reveals and reports for the first time that a rough morphotype *C. auris* strain causes urinary tract infections in non-intensive care unit (ICU) inpatients. Furthermore, the morphology, the scanning electronmicroscopy (SEM), Whole-genome resequencing and RNA sequencing of *C. auris* possessing rough morphotype colonies compared to their smooth morphotype counterparts.

**Results:**

The newly identified phenotypic variation of *C. auris* appears round, convex, dry, and burr-like with a rough texture. SEM shows that rough type *C. auris* has a rough and uneven colony surface with radial wrinkles and irregular spore arrangement. Cells of the rough morphotype *C. auris* naturally aggregate into clusters with tight connections in the liquid, and it seems that the cell division is incomplete. A genome-wide analysis of the rough type *C. auris* confirmed its genetic association with the smooth type of *C. auris* prevalent in China (Shenyang) two years ago; however, single nucleotide polymorphism (SNP) mutations of five genes (*ACE2*, *IFF6*, *RER2*, *UTP20*, and *CaO19.5847*) were identified more recently. RNA-seq revealed *IFF2/HYR3*, *DAL5*, *PSA31*, and *SIT1* were notably up-regulated, while multiple cell wall-associated genes (*ALS1*, *MNN1*, *PUL1*, *DSE1*, *SCW11*, *PGA38*, *RBE1*, *FGR41*, *BGLI*, *GIT3*, *CEP3*, and *SAP2*) were consistently down-regulated in rough morphotype *C. auris*.

**Discussion:**

The rough phenotypic variation of *C. auris* is likely to be related to the structural and functional changes in cell wall proteins. This novel rough morphotype *C. auris* will provide a basis for further studies concerning the evolutionary characteristics of *C. auris*.

## Introduction

*Candida auris* is a newly emerging pathogenic fungus of global concern. On October 25, 2022, the World Health Organization (WHO) released a list of the WHO fungal priority pathogens (WHO FPPL)^1^. Among them, *C. auris* was ranked in the Critical group as one of the most health-threatening fungi. *C. auris*, an aggressive and drug-resistant type of yeast, has high outbreak potential (Eyre et al., 2018), and this fungus is notoriously difficult to diagnose via using conventional techniques. To make matters worse, it has been suggested that *C. auris* can exhibit three colony types on CHROMagar: (1) pink, (2) white, and (3) sectored (dark purple), and varying amounts of phenotypic switching that can potentially interfere with correct identification (Bentz et al., 2018). However, *C. auris* colonies that have been reported were smooth and glossy without textural changes. In this study, we present a description of a rare case of *C. auris* with rough morphotype colonies. Further in-depth study of phenotypic characteristics and microscopic characteristics using scanning electron microscopy (SEM) will help to enrich our understanding of the morphological characteristics of *C. auris*.

Given that *C. auris* that we previously studied were smooth morphotype colonies (Tian et al., 2018, 2021), we asked whether these natural variant strains of *C. auris* with rough colonies evolved independently with smooth colonies and what else is unique about their genetics. These questions are very interestingly scientific queries. Thus, this study intendes to conduct comparative genomics studies between rough and smooth morphotype of *C. auris* that had been isolated in the previous study.

Moreover, we still lack molecular mechanistic knowledge about the *C. auris* species phenotypic diversification and clinical heterogeneity. Since gene expression variability impacts phenotypic plasticity (Jenull et al., 2021), we aim to characterize transcriptomic signatures of *C. auris* isolates with a distinct phenotype (rough morphotype colonies versus smooth morphotype colonies). This study will advance our understanding the genetic characteristics and species diversification of *C. auris*.

## Materials and methods

### First case of *C. auris* with rough morphotype colony

An 86 years-old man was hospitalized in the respiratory infection ward (RI) on October 31, 2018 with acute exacerbation of chronic obstructive pulmonary disease (COPD) that was further complicated by respiratory failure. This patient was previously admitted to the respiratory intensive care unit (RICU) from November 4 to 15, 2016.

Due to the patient’s medical history including diabetes mellitus and prostate hyperplasia without indwelling catheterization, the urinanalysis result was notable for a WBC 909.09/HPF (Reference range: 0.11–2.83/HPF) on the date of RI admission. In addition, the WBC count was 6.65 × 10^9^/L (Reference range: 3.5–9.5 × 10^9^/L), the ratio of neutrophils was 63.8% (Reference range: 40–75%) and the blood CRP was 3.8 mg/L (Reference range: 0–5 mg/L), respectively. Cefotaxime sulbactam (4.5 g, twice a day) was administrated empirically for 2 weeks considering the concomitant urinary tract infection. On November 7th, there was no significantly reduction of WBC count according to the urinary examination. The urine bacterial culture was negative; however, the rough morphotype colony of *C. aruis* was isolated on the Sabouraud Dextrose Agar (SDA) plates for the first time and the colony count was approximate 1 × 10^3^. Physician suspected that *C. auris* colonies as small amount of fungi might be colonization or environmental contamination, so no special treatment was administrated targeting the *C. auris* colonies. The antibiotics were adjusted to Cefoperazone sulbactam (1 g, three times a day) or Levofloxacin (500 mg, one a day) for 7 18 days but showed no significant improvement. During the treatment, the urianalyses were ordered multiple times and the WBC results were still elevated (117.38–5602.05/HPF). Multiple midstream clean-catch urine cultures were negative for bacteria, but determined *C. auris* with rough morphotype colony with a colony count of 10^2^∼10^3^ colony forming units (cfu)/mL on Days 7, 17, 18, 19, 20, and 24 (Shown in Table 1). The CRP was 5.5 mg/L on November 11th and the serum (1, 3)-β-D-glucan level was <10 pg/mL on November 18th 2018, respectively. On November 24th, 2018 (Day 24), antifungal therapy was initiated with oral voriconazole (200 mg, twice daily). Four consecutive negative urine cultures were noted on day 27 and the urinary leukocytes were significantly reduced (9.95–11.96/HPF). This patient was then discharged on Day 36.

**TABLE 1:** Urine culture and urinalysis of this patient presenting with *Candida auris* infection during hospitalization.

		Urine culture			Urinalysis						
Admission time	Date	Bacteria	Fungi	Colony count cfu/mL	WBC/ HPF	Yeast	BACT/ HPF	NIT	PH	Leu/ul	SG
Day 1	2018/10/31				**909.09**	**+++**	10	Negative	5.5	500	1.028
Day 4	2018/11/04				**117.38**		5	Negative	6	500	1.022
Day 6	2018/11/06	Negative			**796.29**	**+**	19	Negative	5.5	500	1.028
Day 7	2018/11/07		**rough-type *C. auris* (A112)**	**10^3^**	**909.09**	**+**	68	Negative	6	500	1.024
Day 8	2018/11/08	Negative			**346.42**	**+**	13	Negative	6	75	1.023
Day 9	2018/11/09				**1487.36**		22	Negative	6	500	1.027
Day 10	2018/11/10				**324.02**		5	Negative	6	500	1.025
Day 11	2018/11/11	Negative			**495.62**		3	Negative	6.5	500	1.023
Day 14	2018/11/14				**1273.44**		8	Negative	5.5	500	1.025
Day 15	2018/11/15	Negative			**909.09**	**+**	6	Negative	6	500	1.028
Day 17	2018/11/17	Negative	**rough-type *C. auris* (Unkept)**	**10^3^**	**491.11**		6	Negative	7	500	1.024
Day 18	2018/11/18	Negative	**rough-type *C. auris* (A114)**	**10^3^**	**5602.05**		57	Negative	7.5	500	1.02
Day 19	2018/11/19	Negative	**rough-type *C. auris* (Unkept)**	**10^3^**	**909.09**	**+**	53	Negative	7	500	1.021
Day 20	2018/11/20	Negative	**rough-type *C. auris* (Unkept)**	**10^2^**	**163.49**		1	Negative	7	75	1.018
Day 21	2018/11/21				**1310.44**		4	Negative	5.5	500	1.019
Day 24	2018/11/24		**rough-type *C. auris* (Unkept)**	**10^3^**	**153.05**		3	Negative	7	75	1.011
Day 27	2018/11/27	Negative	Negative		**9.95**		0	Negative	8.5	Negative	1.013
Day 32	2018/12/02	Negative	Negative		**11.96**		0	Negative	7	Negative	1.013

Meaningful increases were shown in bold values. The result of yeast showed that “+” was < 1/4 per HPF; “+++” was > 3/4 per HPF. WBC, white blood cell; HPF, high-power fields; CFU, colony forming unit; BACT, bacteria; NIT, nitrite; Leu, leucocyte esterase.

### Strains identification and antifungal susceptibility tests

*C. auris* strains were grown in SDA and CHROMagar Candida medium (CHROMagar, Paris, France). Liquid cultures were inoculated in yeast–peptone–dextrose (YPD) liquid medium (1% yeast extract, 2% bactopeptone, 2% glucose). Yeast extract, bactopeptone and glucose were purchased from HopeBio Company. Calcofluor White staining solution was purchased from Sigma. Final concentration of 10 μg/ml Calcofluor White solution was added into cells and incubated for 10 min in dark, cells were observed by fluorescent microscopy. For salt resistance test, *C. aurs* strains were incubated on YPD-agar plates with different NaCl concentrations of 10, 12, 15% (w/v) at 37°C for 24 h∼48 h, and then check the growth. All *C. auris* isolates were identified using the matrix-assisted laser desorption ionization time of flight (MALDI-TOF) (VITEK-MS system) with the RUO database. Antifungal susceptibility testing was performed using the Sensititre YeastOne colorimetric micro-dilution method (Thermo Fisher scientific) as described previously (Tian et al., 2021).

### Scanning electron microscopy

For cultured samples, a scalpel blade was used to remove a whole yeast colony with 5 mm of the surrounding agar from the culture plate as described previously (Radford et al., 1994). The sample was plunged into liquid nitrogen, frozen for 2 min, removed carefully, and then placed on a pre-cooled stainless steel dissection plate. Next, the sample was transferred using a refrigerated transfer system PP3010T (Quorum). The samples were coated with platinum and viewed by SEM (Hitachi Regulus 8100) at 5 kV. For liquid samples, after washing with ddH_2_O, 1 × 10^5^ cells of *C. auris* were dropped into filter paper. The other steps were described above.

### Whole-genome resequencing

Whole-genome sequencing (WGS) was performed using the Illumina NovaSeq platform (by Berry Genomics Co., Beijing, China) as described previously (Tian et al., 2021). The variants that were predicted to alter the protein sequence in any coding sequence [non-synonymous single nucleotide variants (SNVs), stop loss or gain variants, indels] were annotated by ANNOVAR software (Wang et al., 2010) using the RefSeq *C. auris* B11221 coding sequence.^2^

### Phylogenetic analysis *C. auris* with rough morphotype colony

In order to determine the evolutionary relationship between the smooth and rough morphotype of *C. auris*, we selected two rough morphotypes (RI A112 and RI A114) from one patient and previously isolated smooth morphotypes from different patients in our hospital [see previous studies for details (Tian et al., 2021)]. The genetic evolution analysis involved 116 SNP sites of 39 strains. The evolutionary history was inferred by using the maximum likelihood method based on the Tamura-Nei model (Tamura and Nei, 1993). All positions containing gaps and missing data were eliminated. A total of 116 positions in the final dataset were obtained. Evolutionary analyses were conducted in MEGA7 (Kumar et al., 2016). The phylogenetic tree was constructed and composed by iTOL software.^3^

### RNA sequencing

The strains that underwent transcriptomic profiling included two rough morphotype colonies of *C. auris* (RI A112 and RI A114) and a smooth morphotype colony of *C. auris* (RICU A1), which was the first strain identified in Shenyang (China). RNA sequencing (RNA-seq) analysis was performed using the Illumina NovaSeq platform (Berry Genomics Co., Beijing, China). *C. auris* cultures grown overnight were inoculated into YPD medium. Total RNA was purified using a GeneJET RNA purification kit (Thermo Scientific). The quality of RNA was assessed on a bioanalyzer using the RNA6000 Nanochip (Agilent), mRNA was enriched using oligo (dT) beads [New England BioLabs (NEB)], and subsequently, double-stranded cDNA libraries were generated using the NEBNext Ultra directional RNA library prep kit for Illumina (NEB) according to the manufacturer’s instructions. The qualified libraries were subjected to Illumina sequencing with 150 bp paired-end reads at the Novogene sequencing facility. Three biological replicates for each strain were sequenced.

Samples were sequenced on the platform, and the original data in FASTQ format (Raw Data) were generated. In the data mapping analysis, the reference genome (*C. auris* B11221) and gene annotation files were downloaded from genome website. The reference genome index was constructed by Bowtie2 (Langmead and Salzberg, 2012), and the filtered reads were mapped to the reference genome using HISAT2 (Siren et al., 2014). We then used HTSeq (Anders et al., 2015) statistics to compare the Read Count values on each gene as the original expression of the gene, and then used fragments per kilobase of exon model per million reads (FPKM) to standardize the expression. We then used DESeq (Anders and Huber, 2010) to analyze the genes of difference expression with screened conditions consisting of expression difference multiple | log_2_FoldChange| > 1, and a significant adjusted *p*-value < 0.05. At the same time, we used R language Pheatmap software package to perform bi-directional clustering analysis of all different genes of samples. Next, we used topGO to map all genes to terms in the Gene Ontology (GO) database and calculated the numbers of differentially enriched genes in each term based on hypergeometric distribution. In addition, the “annotation module” of the Kyoto Encyclopedia of Genes and Genomes Orthology Based Annotation System (KOBAS) (Bu et al., 2021) was applied to conduct the annotation of some specific genes that have no characterized homolog of *C. albicans* in the Swissprot databases.

## Results

### Microbiological characteristics of *C. auris* with rough morphotype colonies

**FIGURE 1:**
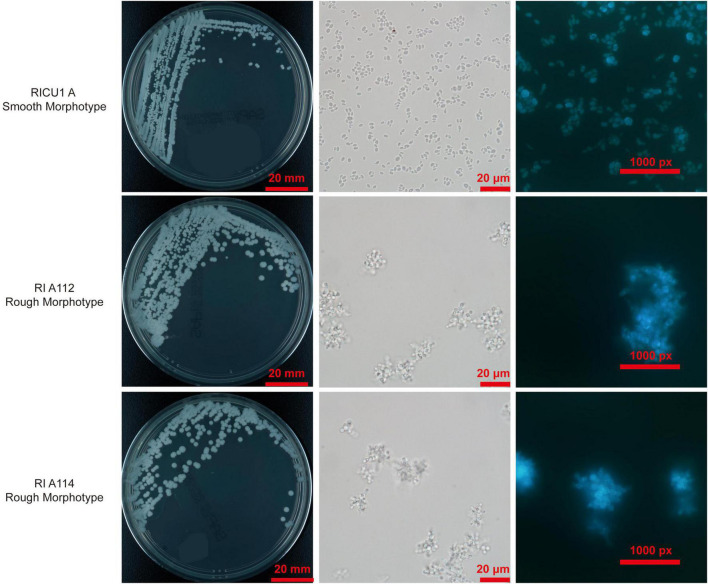
Photo of rough morphotype *Candida auris* colonies grown on Sabouraud Dextrose Agar (SDA) medium and Chromogenic media (CHROM agar), smooth counterparts as controls. The microscopy of the cells of rough morphotype *C. auris* under differential interference contrast (DIC) and the Calcofluor White stain. As described in Materials and methods, final concentration of 10 μg/ml Calcofluor White solution was added into cells and incubated for 10 min in dark, cells were observed by fluorescent microscopy. DIC photos of *C. auris* cells and Calcofluor White stain, smooth counterparts as controls.

On SDA plates, the rough morphotype *C. auris* cultured at 35°C for 24 h and grew 1 mm tiny colonies that appeared round, convex, dry, and burr-like with rough texture. Colonies were uniform after 3 days and gradually grew into white colonies with a central bulge, surrounding wheel-shaped radiation folds, and uneven edges (Figure 1). Rough pink colonies appear on the Chromogenic media (CHROM agar) (Figure 1 R2). As with the smooth morphotype *C. auris*, the rough strain exhibits high temperature resistance (grew well at 28–42°C with weak growth at 45°C) and salt resistance (grew well in 10–12% NaCl, but poorly in 15% NaCl). It was viable in 0.05% cycloheximide. The phenotype of rough type *C. auris* was hereditable and was retained even after 30 passages at room temperature for more than half a year.

Microscopic observation [Differential interference contrast (DIC)] showed yeast cells of the rough morphotype *C. auris* often aggregated into clusters in the natural state (liquid culture is easy to precipitate), appeared as round spores with a chain-like arrangement and close connections among spores (Figure 1). Based on microscopy of Calcofluor white staining, the cells of rough morphotype *C. auris* were found to naturally clump together with tightly connected spores (Figure 1).

All rough morphotype *C. auris* isolates were resistant to fluconazole and sensitive to voriconazole, itraconazole, amphotericin B, and caspofungin (Tian et al., 2021). No differences between all rough morphotype *C. auris* isolates and smooth morphotype *C. auris* isolates were found (Tian et al., 2021).

### Comparison of scanning electron microscopy of *C. auris* with rough morphotype colonies and smooth morphotype colonies

**FIGURE 2:**
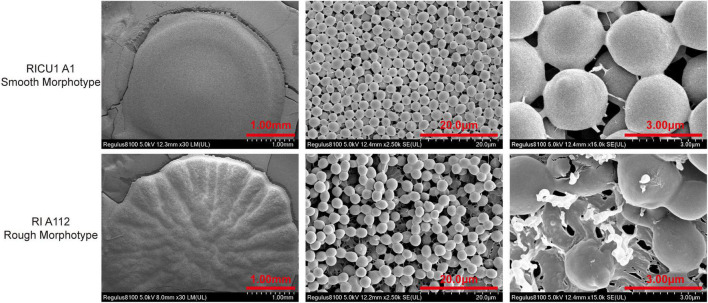
Electron microscopy scan photos with a single *C. auris* colony on Sabouraud Dextrose Agar (SDA) medium, which was cultured at 35°C for 3 days. Frozen transmission (liquid nitrogen) the whole process (rough morphotype Ra/Rb/Rc; smooth morphotype Sa/Sb/Sc). *C. auris* was cultured in Yeast–peptide_dextrpsse (YPD) liquid medium at 35°C for 3 days and then washed in ddH2O, after which 1 × 10^5^ cells of *C. auris* were dropped on filter paper and frozen in liquid nitrogen. Electron microscopy was performed and photos were obtained (rough morphotype Rd/Re/Rf; smooth morphotype Sd/Se/Sf).

To thoroughly identify the differences between rough and smooth morphotype *C. auris*, SEM was employed, which showed that the colony surface of *C. auris* was rough and uneven and had radial wrinkles, and the arrangement of spore was irregular. The cells of the rough morphotype *C. auris* were closely connected in the liquid, and most cells with rough morphotype appearance were connected at septa. It appears that budding daughter cells could not detach from the mother cells, resulting in incomplete division (Figure 2).

### Phylogenetic analysis of 39 clinically isolated *C. auris*

To decipher the evolution of the rough morphotype *C. auris*, we performed phylogenetic analysis with the previously isolated strains (Tian et al., 2021), the results of which showed all *C. auris* from the RICU were related to RICU A1, the first specimen isolated from this facility, and rough morphotype *C. auris* (RI 112 and RI 114) belongs to the same subclade as RICU4 A7, RICU6 A14, RICU5 A12, and RICU17 A108.

**FIGURE 3:**
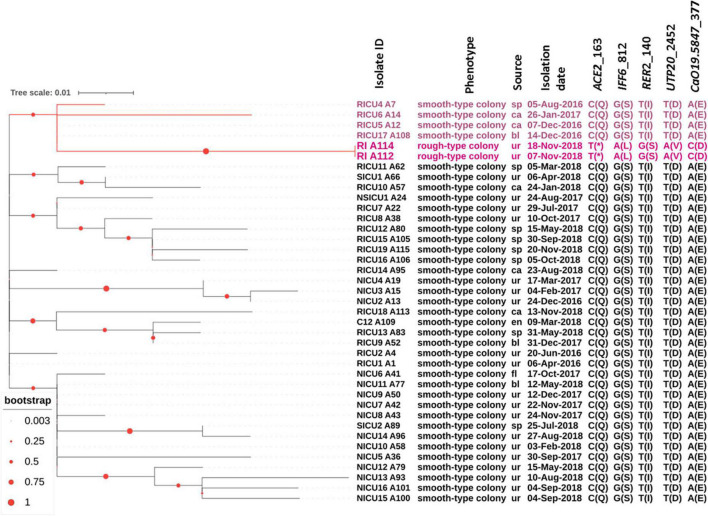
Molecular phylogenetic analysis and other relevant information. All *C. auris* strains were isolated from our hospital. The tree was generated using the maximum-likelihood method using MEGA7. The phylogenetic tree was constructed and composed by iTOL software. Among a total of 39 samples, 37 samples were from the smooth type of *C. auris*, and two were from rough types of *C. auris* (RI A112 and RI A114). The branches and the sample names with rough types of *C. auris* and related closely related ones are shown in red. Each sample corresponds to a mark in the phenotype, source, isolation date, nucleotide mutation site, and amino acid variation in parentheses. RICU, respiratory ICU; NICU, neurosciences ICU; NSICU, neurosurgical ICU; SICU, surgical ICU; RI, respiratory infection.

Interestingly, the RI A112 and RI A114-isolated patient had a medical history in RICU in 2016; at that time, RICU17 and RICU5 patients were also admitted in this department. Thus, we speculated that the rough morphotypes were originated from the smooth morphotypes due to the spatial and temporal crossover, though they were from different patients. According to the phylogenic analysis, we found that the two rough types of *C. auris* (RI_A112 and RI_A114) were the most closely genetically associated with the smooth type of *C. auris* (RICU17_A108 and RICU5_A12), which were isolated in December, 2016. This finding corresponds to our “spatial time crossover” hypothesis, suggesting that rough types of *C. auris* may be evolutionarily close to the smooth type of *C. auris*. The above data support that *C. auris* with rough type colonies most likely originated from the smooth type of *C. auris* rather than having an independent origin (Figure 3).

In the whole genome sequence analysis of *C. auris* with rough morphotype colonies versus smooth morphotype colonies, five genes were found to have non-synonymous mutations. The proteins encoded by five genes corresponding to the homologues of *C. albicans* were Ace2, Iff6, Rer2, Utp20, and CaO19.5847 (see Figure 3 for details). In another study, *CauACE2* was experimentally verified as a key regulator of morphogenesis, and the Δ*ace2* mutant of *C. auris* resulted in constitutively aggregating cells with individual cells connected at septa, suggestive of a failure of budding daughter cells to separate from the mother cells (Santana and O’Meara, 2021). This process is strikingly similar to the natural aggregating phenotype of *C. auris* with rough morphotype colonies in this study. Identification Friend or Foe (IFF)6 indicated putative glycosylphosphatidylinositol (GPI)-anchored proteins (Boisrame et al., 2011) and is predicted to be an essential gene encoding for an adhesin-like cell wall protein. For *C. albicans*, cis-prenyltransferase Rer2 is required for protein glycosylation, cell wall integrity, and hypha formation (Juchimiuk et al., 2014). Utp20 is a putative snoRNA-binding protein and is likely essential for growth (Ev et al., 2020).

### Comparative transcriptional profiling of *C. auris* with rough morphotype colonies vs. smooth morphotype colonies

**FIGURE 4:**
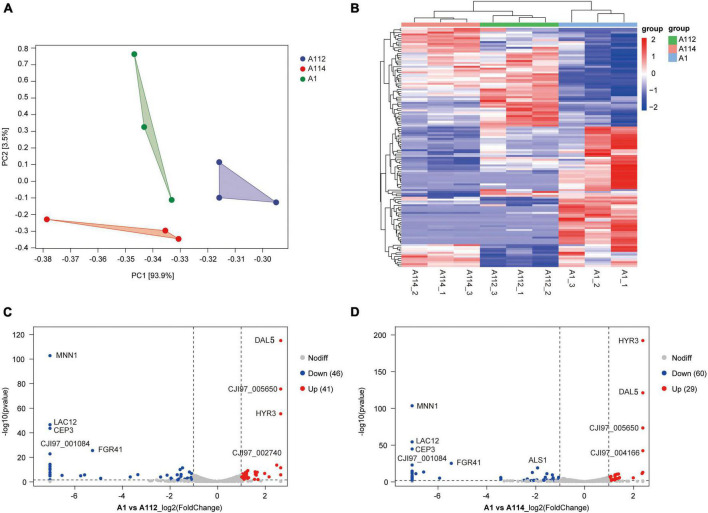
Pairwise differential expression analysis of A1 vs. A112, and A1 vs. A114. **(A)** Principal component analysis (PCA) of fragments per kilobase of exon model per million reads (FPKM) profiles in rough-type colony vs. smooth-type colony groups. **(B)** Cluster analysis of patterns of gene expression in rough-type colony vs. smooth-type colony groups. **(C)** Volcano plot of pairwise differential expression analysis of A1 vs. A112. Fold-change (log_2_) in A1 versus A112 was plotted against the false discovery rate (FDR). Red dots indicate upregulation and blue dots indicate down-regulation. **(D)** Volcano plot of pairwise differential expression analysis of A1 versus A114. Fold-change (log_2_) in A1 versus A114 was plotted against the FDR. Red dots indicate upregulation and blue dots indicate downregulation.

Firstly, we conducted Principal component analysis (PCA) and Cluster analysis of gene expression in rough-type colony vs smooth-type colony groups (Figures 4A, B). Secondly, we performed the GO and KEGG analyses for differential expression genes, which showed a significant enrichment in membrane associated GO terms (Supplementary Figure 1). And then, we performed a pairwise comparison of A1 vs. A112 and found a limited set of 41 upregulated genes and 46 downregulated genes (Figure 4C). while the pairwise comparison of A1 vs. A114 showed 29 upregulated genes and 60 downregulated genes (Figure 4D). The highest upregulated genes included a GPI-anchored cell wall protein Iff2/Hyr3 (Boisrame et al., 2011) and two transmembrane transporters homologous to *Saccaharomyces cerevisiae* DAL5, which encodes for allantoate permease (Hellborg et al., 2008) and SIT1/ARN1, which encodes for a siderophore iron transporter (Heymann et al., 2002). The most common downregulated genes included the homolog of the *C. albicans MNN1*, encoding for an alpha-1, 3-mannosyltransferase (Bates et al., 2013), and the homolog of *Kluyveromyces lactis LAC*12, encoding for lactose permease. In addition, the homologues of *S. cerevisiae CBF3* were found to encode for centromere DNA-binding protein complex, and the homologues of *C. albicans FGR41*, as effector gene in the calcineurin regulon, encode for putative cell wall proteins and mediate masking/unmasking (Wagner et al., 2022).

**FIGURE 5:**
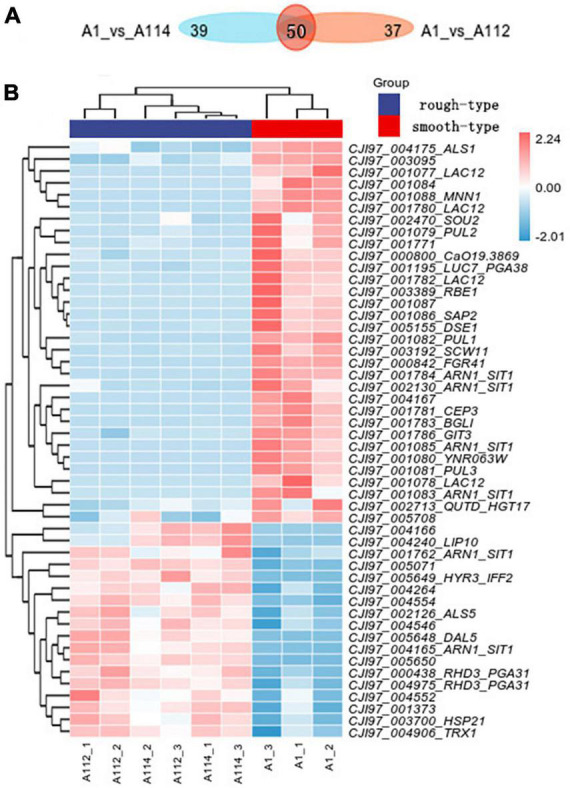
Transcriptomic profiling of rough morphotype colony of *C. auris* (A112 and A114) vs smooth morphotype colony of *C. auris* (A1). **(A)** Venn diagram of the number of differentially expressed genes (DEGs) in rough morphotype colony versus smooth morphotype colony groups. **(B)** A heatmap of centered and scaled fragments per kilobase of exon model per million reads (FPKM) values of DEGs in rough morphotype colony versus smooth morphotype colony groups.

A112 and A114 shared 50 differentially expressed genes in common when compared to the smooth colony group, 18 of which were upregulated and 32 of which were downregulated (Figures 5A, B).

Among them, the top upregulated genes encompassed the key cell wall proteins, including two homologues of the *C. albicans PGA31*, linked to chitin assembly during cell wall biogenesis and the maintenance of wall integrity under cellular stress (Plaine et al., 2008).

Most interestingly, two homologues of *C. albicans* SIT1/ARN1 were upregulated, and four copies were downregulated.

On the other hand, many genes encoding cell wall-related proteins were found to be downregulated (*ALS1*, *MNN1*, *PUL1*, *DSE1*, *SCW11*, *PGA38*, *RBE1*, *FGR41*, *BGLI*, *GIT3*, *CEP3*, and *SAP2*) as shown in Figure 5B. Among them, we noted an interesting phenomenon in this study in which the top downregulated genes, such as homologues of *C. albicans MNN1* (Bates et al., 2013) and *SAP2*, encode for a secreted aspartic protease (Naglik et al., 2003), and in another study, these genes were strikingly upregulated in an azole-resistant isolate of *C. auris* when compared with the azole-sensitive isolate of *C. auris* (Jenull et al., 2021). These findings suggest that the two genes may play different roles in regulating differences in drug resistance and phenotypic variation. Moreover, there are four copies of *LAC12* in downregulated gene, and the latter has no homologous gene in *Candida* spp. so its biological function is unknown. In addition, 14 of them have no characterized homolog in the databases.

## Discussion

We noted a case of *C. auris* with an unusual rough morphotype colony phenotype. Different from previous reports of smooth colonies of *C. auris* (Bentz et al., 2018; Tian et al., 2018), the colony surface of this newly identified *C. auris* showed a rough texture and was uneven with radial wrinkles. Without *in vivo* induction through a mammalian host (Yue et al., 2018), the phenotypic switch of rough morphotype *C. auris* occurs naturally and is stably inherited even after dozens of generations. For previous smooth morphotype *C. auris* in its natural state, microscopic examination showed round or oval spores with a scattered arrangement, and after 1 min of vortex mixing, some cells could aggregate into clumps, which were not easy to disperse as described in other studies (Borman et al., 2016). The cells of rough morphotype *C. auris* under the action of no external force clustered together, were arranged in chains, were closely connected between the spores, and naturally aggregated into large clusters in the liquid. Under SEM, we found that most cells with rough morphotype appearance were connected at the septa, and it appeared that budding daughter cells could detach from the mother cells, resulting in incomplete division. As a result, the appearance resulted in a rough colony. Genome-wide comparisons of *C. auris* with rough and smooth type colony morphologies revealed an amino acid mutation in *CAUACE2*, a key regulator of morphogenesis (Santana and O’Meara, 2021), has a specific SNP variation and amino acid mutation. Further experiments are needed to confirm whether this particular mutation is associated with the emergence of rough colonies in *C. auris*.

We speculated that the smooth-rough morphological changes of *C. auris* appear to be related to cell wall structure and functions. Comparative transcriptional profiling of *C. auris* with rough morphotype colonies versus smooth morphotype colonies showed that *IFF2/HYR3* [GPI-anchored cell wall protein (Boisrame et al., 2011)], *DAL5* [encoding for the allantoate permease (Hellborg et al., 2008)], *PSA31* [the key GPI-associated cell wall gene (Plaine et al., 2008)], and *SIT1* [iron metabolism-associated gene (Heymann et al., 2002)] were strikingly upregulated in rough morphotype *C. auris*. Among them, *PGA31* was reported closely related to chitin assembly during cell wall biogenesis and the maintenance of cell wall integrity under stress (Plaine et al., 2008). Moreover, another study described similar results in which *PGA31* was upregulated in filamentous cells (Yue et al., 2018). Furthermore, in recent studies, the monoclonal antibodies prepared by Psa31 could lead to a significant improvement in the survival rate in a series of clinically predictive mouse models of systemic candidiasis of *C. albicans*, which is expected to be a potential target for therapeutic antibody (Palliyil et al., 2022). In addition, the homologues of *C. albicans* Hsp21, the novel small heat shock protein, mediates stress adaptation and virulence in *C. albicans* (Mayer et al., 2012). The top upregulated genes also include the homologues of *C. albicans TRX1*, encoding for Thioredoxin-1 that can respond specifically to oxidative stress (Enjalbert et al., 2007). Another study confirmed that siderophore uptake mediated by Sitp/Arn1p is required in a specific process during the infection process of humans, namely the infection of epithelial layers (Heymann et al., 2002). Therefore, the function of these genes deserve more attention and further study.

At the same time, many cell wall-related protein coding genes were downregulated. These data strongly supported that the rough phenotypic variation of *C. auris* is likely to be related to the structural and functional changes in cell wall proteins. Recent studies have shown that switched morphotypes colonies of *C. tropicalis* have distinct expressions of the cell wall-associated master regulators genes (de Souza et al., 2022).

Furthermore, we tried to trace the origin of the rough morphotype *C. auris* and were surprised to find that it seemed to be related to the previous smooth morphotype *C. auris.* The genome-wide analysis of two rough morphotype *C. auris* had confirmed that it derived from the same lineage as the smooth morphotype *C. auris* strain isolated from patients who were admitted to the RICU 2 years ago (Tian et al., 2021). This finding should be related to the fact that the patient was once hospitalized in RICU (smooth morphotype *C. auris* was prevalent in this ICU at that time), and in subsequent follow-up, yeast-like cells had been detected three times (January 26, 2017; October 1, 2017; November 6, 2017) in routine urinalysis. Unfortunately, no urine culture was performed. As a result, we only presumed that this patient may have had *C. auris* colonization for 2 years, a finding that is very similar to another recent study in which *C. auris* can cause infections after colonizing the human body for a long time (Heath et al., 2019). Another study confirmed that significant cell aggregation of *C. auris* in the kidneys of mice with hematogenous disseminated candidiasis occurs, suggesting that the characteristics of cell aggregation may help *C. auris* escape host immunity and persist in host tissues (Ben-Ami et al., 2017). As we know, morphological plasticity is a common strategy adopted by fungal pathogens and allows rapid adaptation to a harsh environment, survive and thrive in certain host niches. If *C. auris* colonize within the kidney and urinary system of this patient for 2 years, then to adapt to the high osmotic pressure and high salt environment, a smooth–rough phenotypic transition had very likely occurred. Of course, this is just speculation and needs to be confirmed in animal models in which rough morphotype *C. auris* would be beneficial to immune escape and long-term colonization.

In addition, we described in detail a case with a low-grade disease manifesting as a urinary tract infection caused by *C. auris* with rough morphotype colonies. In general, *C. auris* strains isolated from urine specimens are usually considered to be asymptomatic urogenital colonization (Govender et al., 2018). However, this patient was diagnosed with urinary tract infection caused by *C. auris*, based on several findings: (1) several urine routine tests indicated continuous elevated leukocytes, (2) multiple midstream urine cultures were negative for bacteria and systematic antibacterial treatment failed, (3) detection of *C. auris* in six urine cultures, and (4) the fact that antifungal therapy was effective. Moreover, diabetes mellitus and prostate hyperplasia in elderly patients lead to an increase in the risk of developing urinary tract infections. Since *C. auris* is known for persistent colonization, colonization is often a prerequisite for infection. Clinicians should continuously monitor the status of patients who present with long-term colonization of *C. auris* and be vigilant against the occurrence of *C. auris* infection.

Limitations in our study should be discussed. Firstly, only two strains of *C. auris* with rough morphotype colonies were included in the analysis. Secondly, only a few genes displayed SNPs in the comparative genomic analysis. Thirdly, in order to avoid any deletion of genetic items, we set thresholds (| log_2_FoldChange| > 1 and adjusted *p*-value < 0.05) in accordance with other literature (Wu et al., 2022; Zhou et al., 2022; Gong et al., 2023). Nonetheless, only a dozen genes were changed in the transcriptomic comparison analysis. From another point of view, we should make good use of this special model and try our best to elucidate the key factors affecting the phenotypic switching of *C. auris* from the limited set of variation factors so as to better promote the morphological study of *C. auris*.

In summary, the unexpected rough morphotype *C. auris* phenotype should be noted since it is suggestive that *C. auris* had latent phenotypic plasticity and was undergoing adaptive evolution. We should continue to enrich the study of morphological and genetic characteristics of *C. auris*, so as to help clinicians timely and accurately diagnose diseases caused by *C. auris*.

## Data availability statement

The datasets presented in this study can be found in online repositories. The names of the repository/repositories and accession number(s) can be found in the article/Supplementary material.

## Ethics statement

The studies involving human participants were reviewed and approved by the Ethics Review Committee of the First Affiliated Hospital of China Medical University (ERC number 2019-53-2). Written informed consent for participation was not required for this study in accordance with the national legislation and the institutional requirements.

## Author contributions

ST, JB, YC, HL, and QW have made substantial contributions to conception and design, acquisition of data, or analysis and interpretation of data. SC and JC have been involved in drafting the manuscript. HS have given final approval of the version to be published. All authors contributed to the article and approved the submitted version.

## Footnotes

1
https://www.who.int/publications/i/item/9789240060241

2
http://www.candidagenome.org/

3
https://itol.embl.de
