# Supplementary figures and images for: Phenotypic and genetic features of a novel clinically isolated rough morphotype Candida auris
Source: Front Microbiol. 2023 Jun 7;14:1174878. doi: 10.3389/fmicb.2023.1174878 (PMC10282645; doi:10.3389/fmicb.2023.1174878)

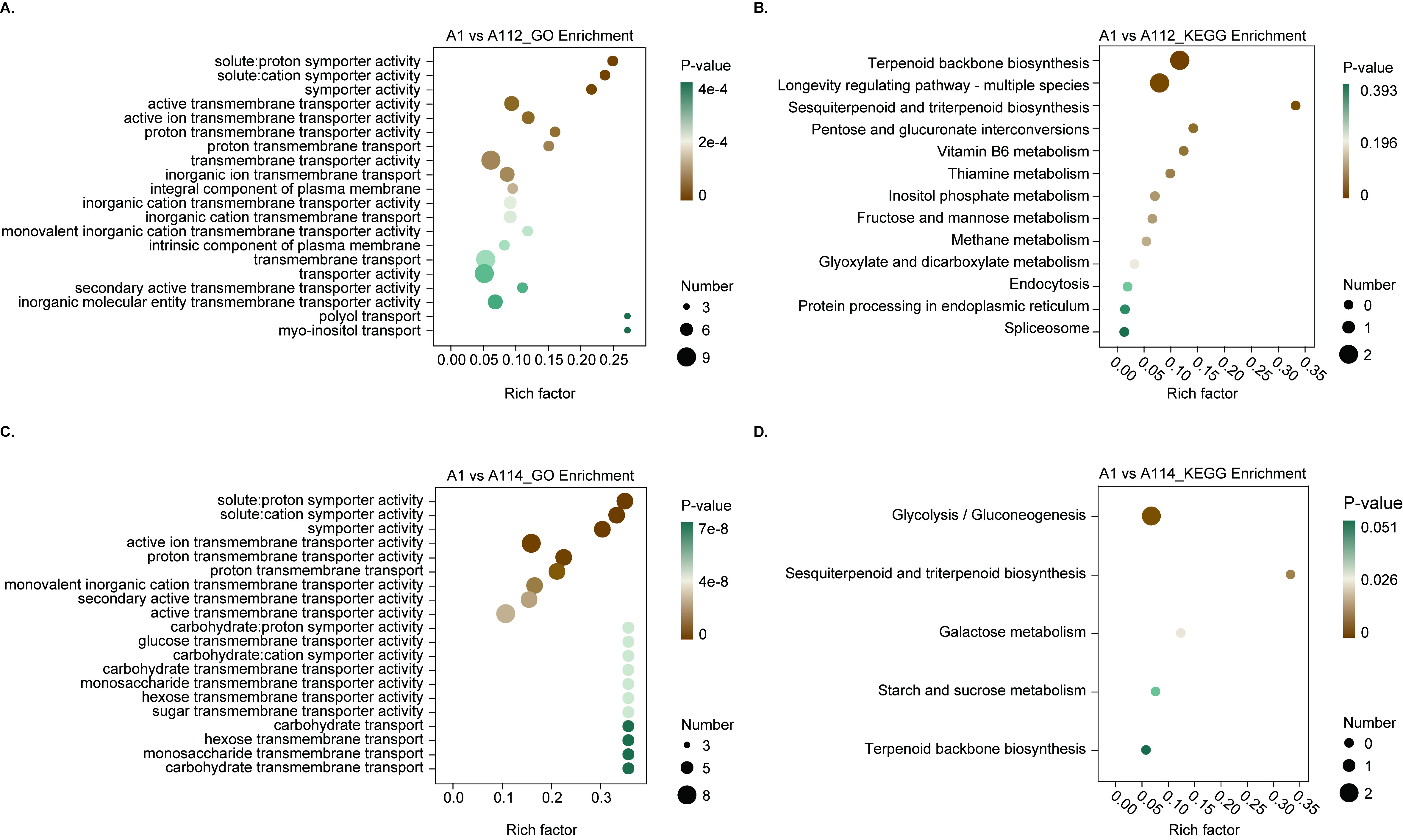

Supplement: Supplementary file 3 [file Image_1.JPEG]
